# Esmarch’s Bloodless Operation

**Published:** 1876-08

**Authors:** F. A. Stanford

**Affiliations:** Chairman of Committee on Surgery, 4th Congressional District


### ESMARCH'S BLOODLESS OPERATION, AS APPLIED TO AMPUTATIONS.

By F. A. STANFORD, M.D.,
Chairman of Committee on Surgery, 4th Congressional District.

From observations made upon a single case of the above operation, as applied to amputations, I am clearly of the opinion that a few objections may be urged against it:

1st. From the immense pressure exerted by the elastic rubber tubing upon the soft tissues, contusion is thereby produced to the extent of wounding the soft parts immediately beneath, and within its line of application down to the periosteum.

2d. As succeeding to the contusion of tissues consequent upon this, oozing of blood takes place from the entire cut surface after the first reaction of the patient, amounting to hemorrhage, necessitating the application of ligatures to a large extent of muscular tissue.

3d. A contused wound inflicted, a solution of continuity is the legitimate result, terminating in suppuration in the effort at repair and reorganization.

4th. The contused wound involves the whole incised or amputated surface in its destructive operations, thereby defeating all effort at adhesive inflammation, which often occurs after amputations after the ordinary method.

5th. From this extensive solution of continuity, retraction of flap to an incalculable extent may follow, and what might have appeared a redundancy of flap at the completion of the amputation. When the granulating process is near completion, a conical stump is difficult to prevent.

Pettit's tourniquet well applied, as a substitute for the rubber tubing, would fully answer the purpose of making it a bloodless operation. There would then be no risk of contusion of the soft parts. The elastic roller I regard an acquisition to surgery.

P. S.—The above report was intended for the State Medical Association, recently convened at Augusta, Georgia, but reached its destination too late to be placed before that body.

